# Evaluation of injectable platelet-rich fibrin produced by a simple twice-centrifugation method combined with vacuum sealing drainage technology in the treatment of chronic refractory wounds

**DOI:** 10.3389/fbioe.2022.979834

**Published:** 2022-10-28

**Authors:** Xin Xue, Yuling Bian, Meng Yang, Wei Wei, Lingmin Meng, Qingfu Zhang, Jianguang Tao

**Affiliations:** ^1^ Department of Burn and Plastic Surgery, The First Hospital of Hebei Medical University, Shijiazhuang, China; ^2^ Department of Obstetrics and Gynecology, Hebei Water Conservancy Hospital, Shijiazhuang, China; ^3^ Department of Burn and Plastic Surgery, Handan Central Hospital, Handan, China

**Keywords:** injectable platelet-rich fibrin, vacuum sealing drainage, chronic refractory wounds, wound inflammation, treatment

## Abstract

**Objective:** To evaluate the effects of injectable platelet-rich fibrin (i-PRF) produced by a simple twice-centrifugation method combined with vacuum sealing drainage on wound inflammation and scar formation in chronic refractory wounds (CRW).

**Methods:** A total of sixty-eight patients with CRW who were admitted to our hospital were enrolled in this study. They were then randomly divided into the study group (*n* = 34) with being treated using negative pressure sealing and drainage technology, and the control group (*n* = 34) with being treated using injectable platelet-rich fibrin in conjunction with negative pressure sealing and drainage technology. The following were the primary outcomes: scar conditions at 1 and 3 months after the wound was fully healed, wound healing time, hospitalization time, wound healing rate, incidence of adverse reactions, serum inflammatory indices, and pain levels were assessed 1 day before treatment and 14 days after treatment. The secondary outcomes were determined by comparing the proportion of positive bacterial cultures in the two groups on the day before therapy, as well as on the seventh and fourteenth days after treatment.

**Results:** The wound healing time and hospital stay in the study group were significantly lower than that in the control group (all *p* < 0.001). The wound healing rate of the study group was significantly higher than that of the control group on the 14th day and 28th day after treatment (all *p* < 0.001). On the 14th day after treatment, the levels of WBC, CRP, and IL-6 in the study group were lower than those in the control group (all *p* < 0.001). The positive rate of bacterial culture in the study group was significantly lower than that in the control group on the 7th and 14th day after treatment (all *p* < 0.05). At 1 month and 3 months after treatment, the VSS score in the study group was lower than that in the control group (all *p* < 0.001). The total defect rate of the study group was also significantly lower than that of the control group (5.88% vs. 29.41%, *p* = 0.011).

**Conclusion:** The i-PRF produced by simple twice-centrifugation method combined with VSD could reduce wound inflammation and improve scar formation in patients with CRW.

## Introduction

Chronic refractory wounds usually refer to wounds that cannot be completely healed in a predictable time according to normal biological steps after conventional treatment due to various factors (Dong et al., 2018). The mechanism of chronic refractory wounds is complex, and the treatment cycle is long. Under normal circumstances, when skin injury occurs, it will be healed through four stages of orderly and cross-influenced bleeding, inflammation, granulation tissue formation, or tissue remodeling (Han and Ceilley, 2017). However, some wounds will stagnate in a certain healing stage or fail to complete the healing process under the interaction of internal and external causes, and eventually form chronic refractory wounds (Wang et al., 2020). The commonly used treatment methods for chronic refractory wounds include wound dressing change and debridement surgery, but these methods can only remove necrotic tissue from the surface of the wound and control local infection, with no obvious advantages in reconstructing blood supply, promoting the secretion and activity of growth factors, and controlling excessive apoptosis of cells (Akita et al., 2019)^.^ Therefore, finding new therapies to promote chronic refractory wound healing has become a research hotspot.

Injectable platelet-rich fibrin (i-PRF) is new biotechnology for stimulating and accelerating tissue healing. It has been widely used in plastic surgery and oral surgery in recent years (Vares et al., 2021). The i-PRF is a concentrated solution of platelet extracted from whole blood by centrifugation, including the release of a large number of growth factors and white blood cells such as lymphocytes, neutrophils, and monocytes, which can enhance The anti-infection ability of wound surface (Gurger et al., 2021). It was reported that i-PRF had higher contents of platelets, growth factors, cytokines, and white blood cells, and had strong mobility and anti-bacterial activity (Karde et al., 2017). Vacuum sealing drainage (VSD) is a creative combination of closed dressing and wound drainage technology, which has been widely used in various chronic wounds of multiple disciplines, but it still has limitations in treating some complex chronic wounds and promoting wound tissue regeneration (Lalezari et al., 2017). Considering that the healing of chronic refractory wounds is affected by both internal and external factors, the combination of i-PRF and VSD is a positive treatment plan for chronic refractory wounds. However, there are still few clinical reports on the efficacy of this treatment scheme. Based on this, this study aimed to evaluate the effects of i-PRF combined with VSD on wound inflammation and scar formation in patients with chronic refractory wounds. This study developed a simple twice-centrifugation method to obtain the i-PRF and used the i-PRF in conjunction with negative pressure sealing and drainage technology to treat patients with chronic refractory wounds, reducing the wound surface, inhibiting the inflammatory response, and reducing the degree of scar formation.

## Materials and methods

### Study patients

In this prospective, a total of 68 patients with chronic refractory wounds admitted to our hospital from September 2020 to September 2021 were included, and they were randomly divided into study group and control group according to the random number table method, 34 patients in each group.

Inclusion criteria: 1) chronic and refractory wounds caused by various reasons; 2) patients with no signs of improvement or aggravation after conventional dressing change debridement treatment for more than 4 weeks; 3) patients who refused flaps or skin grafts and received i-PRF or VSD; 4) bacterial and fungal cultures of venous blood were negative; 5) patients who signed informed consent forms. Exclusion criteria: 1) patients who had a history of taking anticoagulants and immunosuppressants 2 weeks before admission; 2) patients with blood disorders; 3) patients whose wound was caused by a malignant tumor; 4) patients with severe heart or lung diseases who cannot tolerate surgery and anesthesia; 5) patients with neurological disorders; 6) patients with wound area >100 cm^2^ and severe gangrene requiring amputation; 7) patients with bacteremia and sepsis.

The study followed the tenets of the Declaration of Helsinki and was approved by the ethics committee of our hospital, and the informed consent forms were obtained from all patients.

### Debridement procedure

Considering that chronic refractory wound is a chronic wasting disease, patients with anemia, electrolyte imbalance, hypoproteinemia, and other complications should be actively corrected before surgery. Patients with mild comorbidities received nutritional support through diet, and patients with severe comorbidities received an infusion of blood products and fluid rehydration treatment (Chen et al., 2021).

First, general anesthesia or nerve block anesthesia was selected according to the location of the wound. Hydrogen peroxide solution, iodophor disinfectant solution, and normal saline were used to wash the wound three times. Then the necrotic tissue and secretions around the wound were removed to thoroughly clean the necrotic tissue in the basal layer of the wound and the lacunae. If necessary, the wound was enlarged and the wound was fully exposed. In the process of curettage, it is necessary to avoid damaging the nerves and blood vessels around the wound. After thorough debridement, a continuous pressure pulse was used to wash until there was no necrotic tissue, purulent secretions, or foreign bodies in the wound surface and surrounding lacunae. Finally, the wound was irrigated with normal saline containing gentamicin (4 × 10^5^ U/250 ml) and the wound was filled with infiltrating gauze for 10 min.

### Procedure of injectable platelet-rich fibrin and VSD

1) Preparation of i-PRF

**FIGURE 1:**
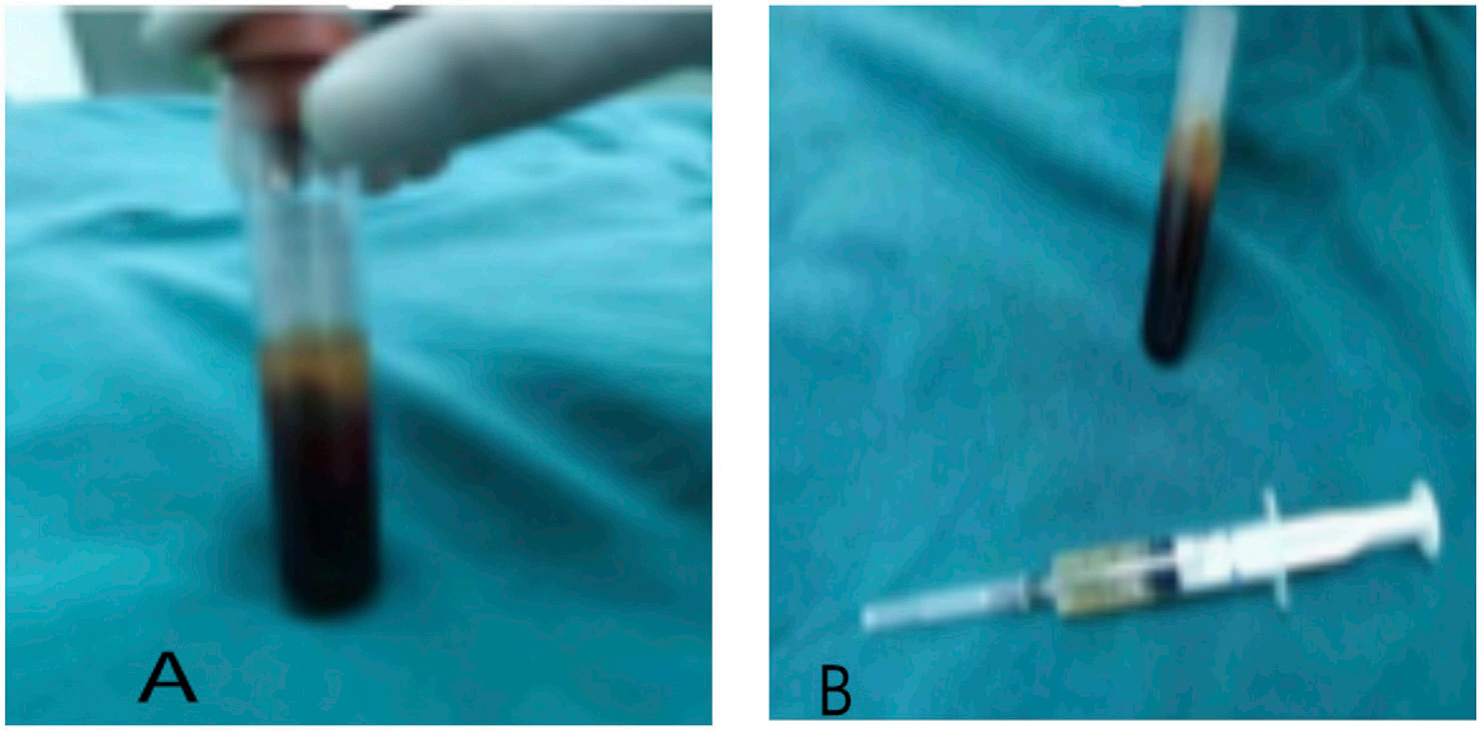
The preparation of injectable platelet-rich fibrin by the twice-centrifugation method. **(A)**: The status of blood samples after the first centrifugation. **(B)**: The status of blood samples after the second centrifugation, and the injectable platelet-rich fibrin has been transferred to a syringe for injection.

**FIGURE 2:**
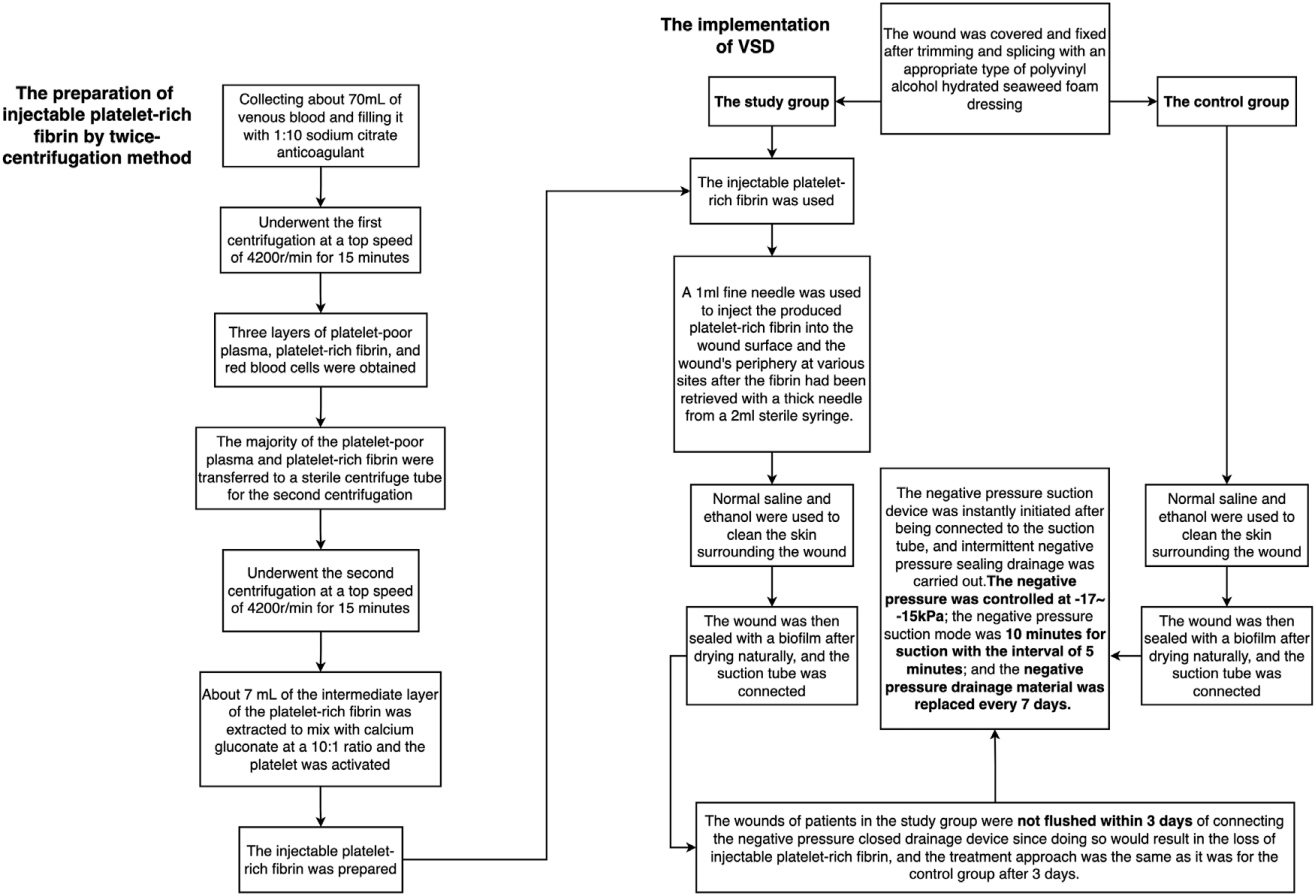
The flow chart of the preparation of injectable platelet-rich fibrin by a twice-centrifugation method and the implementation of VSD.

The injectable platelet-rich fibrin was made using the second centrifugation method, and the aseptic procedure was closely adhered to throughout the entire procedure. Patients who had taken a 30-min nap before lunch had their cubital venous blood taken, and 70 ml of the sample was filled with a 1:10 sodium citrate anticoagulant. Using LC-530 (produced by Shanghai Jiangdong Instrument Co., Ltd.) at a top speed of 4200 r/min for 15 min, the first low-speed centrifugation was performed. Platelet-poor plasma (PPP), platelet-rich fibrin, and red blood cells were the three layers that made up the sample in the tube (Figure 1A). The majority of the liquid in the upper and intermediate layer was transferred using a pipette to a sterile centrifuge tube for the second centrifugation. The sample in the tube was split into three layers following the second centrifugation, which was carried out in the same manner as the first. The bottom layer contained a tiny number of red blood cells, the top layer PPP, and the middle layer platelet-rich fibrin (Figure 1B). About 7 ml of the intermediate layer of platelet-rich fibrin was extracted so that it could mix with calcium gluconate at a 10:1 ratio and activate the platelet. The process of preparing platelet-rich fibrin for injection into wounds is now complete. (Figure 1A,B and Figure 2).

2) The implementation of VSD

The traditional VSD therapy was used to treat the control group. The wound was covered and fixed after trimming and splicing with an appropriate type of polyvinyl alcohol hydrated seaweed foam dressing (Shandong Chuangkang Biotechnology Co., Ltd.) based on the shape and size of the wound. Normal saline and ethanol were used to clean the skin surrounding the incision in turn. The wound was then sealed with a biofilm after drying naturally, and the suction tube was connected. The negative pressure suction device was instantly initiated after being connected to the suction tube, and intermittent negative pressure sealing drainage was carried out. A total of 30 ml of 0.9% normal saline and 160,000 units of gentamicin were used for rinsing each time, and the dressing was changed after each rinse. The negative pressure was controlled at -17-15 kPa; the negative pressure suction mode was 10 min for suction with the interval of 5 min; and the negative pressure drainage material was replaced every 7 days.

**FIGURE 3:**
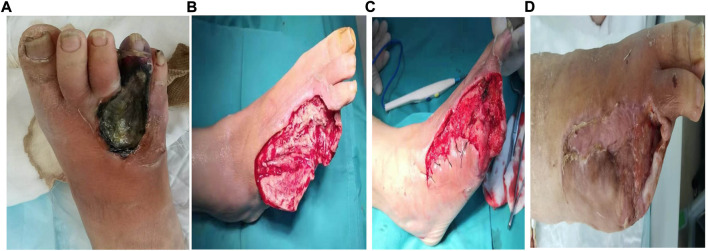
A typical case of the chronic refractory wound cured by the injectable platelet-rich fibrin produced by simple twice-centrifugation method combined with vacuum sealing drainage technology. **(A)** Before the therapy. **(B)** After the debridement, the injectable platelet-rich fibrin was used. **(C)**: The wound condition after 7 days of treatment by the injectable platelet-rich fibrin combined with negative pressure vacuum sealing drainage. **(D)**: The wound condition after 4 months of treatment by the injectable platelet-rich fibrin combined with negative pressure vacuum sealing drainage.

The study group was treated by i-PRF combined with the traditional VSD therapy. A 1 ml fine needle was used to inject the produced platelet-rich fibrin into the wound surface and the wound’s periphery at various sites after the fibrin had been retrieved with a thick needle from a 2 ml sterile syringe. VSD’s setup and installation were identical to those of the control group. The wounds of patients in the study group were not flushed within 3 days of connecting the negative pressure closed drainage device since doing so would result in the loss of i-PRF, and the treatment approach was the same as it was for the control group after 3 days. (Figures 2, 3).

### Evaluation of the therapeutic effect

The following were the primary outcomes: scar conditions at 1 and 3 months after the wound was fully healed, wound healing time, hospitalization time, wound healing rate, incidence of adverse reactions, serum inflammatory indices, and pain levels were assessed 1 day before treatment and 14 days after treatment. The secondary outcomes were determined by comparing the proportion of positive bacterial cultures in the two groups on the day before therapy, as well as on the seventh and fourteenth days after treatment.

Wound healing time, hospital stay, and wound healing rate were recorded. The wound healing standard is that the wound is completely closed, completely covered by epithelial tissue, without dehiscing or ulceration, etc (Liao et al., 2020). The wound healing of the patients was observed on the 14th and 28th day of treatment, and the wound healing rate was calculated according to the measured wound healing volume at different time points. The wound healing rate = (preoperative wound volume - postoperative wound volume)/preoperative wound volume ×100%.

Vancouver Scar Scale (VSS) (Mahar et al., 2021) was used to evaluate scars 1 and 3 months after wound healing. The scoring standard is 0–15 points with 16 grades. The scar was evaluated from four aspects of scar thickness, color, softness, and vascular distribution. The higher the score was, the more serious the scar hyperplasia was. The pain degree was assessed by a numerical rating scale (NRS) (Li et al., 2016). On this scale, 0 is no pain, 1–3 is mild pain, 4–6 is moderate pain and 7–10 is severe pain. One day before treatment and on the 14th day of treatment, 5 ml of fasting venous blood was taken from the patients, and the white blood cell (WBC) count, C-reactive protein (CRP), and interleukin 6 (IL-6) levels were measured after centrifugation.

Bacterial culture was carried out 1 day before treatment and 7 and 14 days after a treatment: first, the wound was washed with normal saline to remove the necrotic tissue, and samples were taken in a “Z” shape. The exudate was evenly dipped from one end of the wound to the other end with a disposable bacterial culture tube and then sent to the laboratory for bacterial culture. Positive rate of bacterial culture = number of positive cases/number of submitted cases ×100%.

Observe whether the inflammatory reaction of the wound surface and abnormal hyperplasia of local granulation tissue occurred during the whole treatment process. The incidence of adverse reactions such as expanded wound area, urticarial, and increased exudation were recorded from the beginning of treatment to 3 months after treatment, and the incidence of adverse reactions was calculated. Incidence of adverse reactions = number of cases of adverse reactions ÷ total number of cases ×100%.

During the whole treatment process, observe whether there is inflammation and abnormal proliferation of local granulation tissue on the wound surface. The adverse reactions that occurred from the start of treatment to 3 months after treatment, such as wound expansion, urticaria, increased exudation, etc., were recorded, and the incidence of adverse reactions was calculated.

### Statistical analysis

The sample size was estimated using the mean comparison method. n_c_=(μ_1-α/2_ + μ_1-β_)^2^s^2^ (1 + 1/k)/(μ_t_−μ_c_)^2^. n_c_ was the number of cases in the control group. μ_1-α/2_ and μ_1-β_ were the percentages corresponding to 1-α/2 and 1-β in the standard normal distribution. T was the mean of the experimental group, c was the mean of the control group, s was the two-group merged standard deviation and k was the proportion of cases in two groups. α = 0.05,β = 0.01,s = $\sqrt{\frac{\left(n1-1\right)s12+\left(n2-1\right)s22}{n1+n2-2}}$ . n_1_ and n_2_ were the number of cases in the two groups. s_1_ and s_2_ were the standard deviations of the two groups. According to the provisions of the China State Food and Drug Administration, 15% was taken as the shedding rate, so the grouped sample size of this study was determined as n = 27 × 1/(1∼0.15) = 70.32 ≈ 70

Statistical analysis was performed using the SPSS software program (version 21.0; IBM Corp, Chicago, IL, United States). Normally distributed measurement data were expressed as mean ± standard deviation (SD), and the comparisons were examined by the Student’s t-test. Categorical variables were presented as numbers with percentages and compared with the χ^2^ test. The test level α was 0.05 on both sides. *p* < 0.05 was considered statistically significant.

## Results

### General data

In this study, a total of 68 patients were finally included. The study group consisted of 14 males and 20 females with an average age of 48.91 ± 7.92 years (ranging from 28 to 59 years). The control group consisted of 16 males and 18 females with an average age of 48.87 ± 7.88 years (ranging from 29 to 58 years). There was no significant difference in age, gender and course of disease between the two groups (*p* > 0.05) (Table 1).

**TABLE 1:** Comparison of general information between two groups.

Index	Study group (*n* = 34)	Control group (*n* = 34)	t/χ^2^	*p* Value
Age (year)	48.91 ± 7.92	48.87 ± 7.88	0.021	0.983
Gender (Male/Female)	14/20	16/18		
Duration (days)	89.21 ± 10.21	89.17 ± 10.65	0.016	0.987
Area of wound (cm^2^)	34.81 ± 9.11	34.78 ± 9.03	0.014	0.989
Type of wound (n)			0.344	0.987
PPWH	6	7		
Traumatic wound	7	6		
Wound of venous ulcer	7	7		
Wound of diabetic foot	11	10		
Pressure ulcer	3	4		
FBG level in patients with diabetic foot (mmol/L)	6.07 ± 1.71	6.01 ± 1.54	0.152	0.879
HbA1c level in patients with diabetic foot	5.62 ± 1.34	5.46 ± 1.59	0.449	0.655
Medicine used by patients with diabetic foot (n)			0.403	0.525
Metformin	2	3		
Repaglinide	5	4		
Insulin	4	3		

PPWH, postoperative poor wound healing; FBG: Fasting blood glucose; HbA1c: glycated hemoglobin.

### Comparison of wound healing time, hospital stay, and wound healing rate

The wound healing time and hospital stay time of the study group were significantly lower than those of the control group (31.19 ± 3.12 days vs. 41.29 ± 4.09 days, *p* < 0.001; 28.98 ± 3.02 days vs. 40.91 ± 3.23 days, *p* < 0.001, respectively). The wound healing rate of the study group was significantly higher than that of the control group on the 14th and 28th days after treatment (71.29% ± 7.38% vs. 65.33% ± 7.31%, *p* = 0.001; 90.12% ± 7.56% vs. 78.91% ± 7.83%, *p* < 0.001, respectively) (Table 2). After treatment, the total defect rate of the study group was significantly lower than that of the control group (Table 3).

**TABLE 2:** Comparison of wound healing time, hospital stay and wound healing rate between two groups.(Mean ± Standard Deviation).

Index	Study group (*n* = 34)	Control group (*n* = 34)	t	*p* Value
Wound healing time (days)	31.19 ± 3.12	41.29 ± 4.09	−11.448	<0.001^a^
Time of hospitalization (days)	28.98 ± 3.02	40.91 ± 3.23	−15.731	<0.001^a^
Wound healing rate (%)				
14th day after treatment	71.29 ± 7.38	65.33 ± 7.31	3.346	0.001^a^
28th day after treatment	90.12 ± 7.56	78.91 ± 7.83	6.006	<0.001^a^

a Compared with the Control group, *p* < 0.05.

**TABLE 3:** Comparisons of the incidence of adverse reactions between two groups, n (%).

Index	Study group (*n* = 34)	Control group (*n* = 34)	χ^2^	*p*
HLGT	0 (0.00)	4 (11.76)		
Local pain aggravation	0 (0.00)	4 (11.76)		
Seepage increase	2 (5.88)	2 (5.88)		
Total defect	2 (5.88)	10 (29.41)	6.476	0.011^a^

HLGT, Hyperplasia of local granulation tissue.

a : Compared with the Control group, p < 0.05.

### Comparison of serum inflammatory markers

Before treatment, there were no significant differences in WBC count, CRP, and IL-6 between the two groups (*p* > 0.05). On the 7th and 14th day after treatment, the WBC count, CRP, and IL-6 in the study group were lower than that in the control group with a significant difference (*p* < 0.001) (Table 4).

**TABLE 4:** Comparison of inflammatory indexes between the two groups (Mean ± Standard Deviation)**.**

Index	Study group (n = 34)	Control group (n = 34)	t	**p Value**
WBC (109/L)				
Before treatment	10.89 ± 2.01	10.81 ± 2.03	0.163	0.871
7th day after treatment	6.32 ± 1.32	7.12 ± 1.38	−5.496	<0.001^a^
14th day after treatment	4.03 ± 1.21	6.21 ± 1.17	−7.552	<0.001^a^
CRP (mg/L)				
Before treatment	8.29 ± 2.06	8.23 ± 2.03	0.121	0.904
7th day after treatment	4.18 ± 1.38	5.38 ± 2.01	−2.869	0.005^a^
14th day after treatment	3.32 ± 1.18	4.23 ± 1.23	−9.955	<0.001^a^
IL-6 (ng/L)				
Before treatment	7.71 ± 1.31	7.68 ± 1.19	0.055	0.956
7th day after treatment	6.29 ± 1.23	6.96 ± 1.11	−10.389	<0.001^a^
14th day after treatment	5.21 ± 1.11	6.22 ± 1.27	−5.475	<0.001^a^

a **:** Compared with the Control group, p < 0.05.

WBC, white blood cell; CRP, C-reactive protein; IL-6, Interleukin 6.

### Comparison of a positive rate of bacterial culture

Before treatment, there was no significant difference in the positive rate of bacterial culture between the two groups (*p* = 0.689). On the 7th day and 14th day after treatment, the positive rate of bacterial culture in the study group was significantly lower than that in the control group (32.35% vs. 58.82%, *p* = 0.028; 5.88% vs. 29.41%, *p* = 0.011, respectively) (Table 5).

**TABLE 5:** Comparison of positive rate of bacterial culture on wound surface between two groups, n (%).

Detection time	Study group (n = 34)	Control group (n = 34)	χ2	p Value
1st day before treatment	30 (88.24)	31 (91.18)	0.159	0.689
7th day after treatment	11 (32.35)	20 (58.82)	4.802	0.028^a^
14th day after treatment	2 (5.88)	10 (29.41)	6.476	0.011^a^

a **:** Compared with the Control group, p < 0.05.

### Comparison of pain and scar scores

Before treatment, there was no significant difference in pain degree and scar score between the two groups (all *p* > 0.05). The pain degree of the study group was lower than that of the control group at 7 d and 14 d after treatment, the difference was statistically significant (all *p* < 0.001). The scar score of the study group was significantly lower than that of the control group at 1 month and 3 months after treatment (*p* < 0.001) (Table 6).

**TABLE 6:** Comparison of pain degree and scar status between two groups (Mean ± Standard Deviation).

Detection time	Study group (*n* = 34)	Control group (*n* = 34)	T	*p* Value
Pain degree				
1st day before treatment	5.82 ± 1.32	5.79 ± 1.29	0.095	0.925
7th day after treatment	3.67 ± 1.22	4.98 ± 0.17	−6.201	<0.001^a^
14th day after treatment	2.67 ± 1.08	3.72 ± 0.13	−5.628	<0.001^a^
VSS score				
1 month before treatment	7.23 ± 1.27	9.06 ± 1.21	−6.083	<0.001^a^
3rd month after treatment	3.28 ± 1.21	5.21 ± 1.27	−6.416	<0.001^a^

VSS, Vancouver Scar Scale.

a Compared with the Control group, *p* < 0.05.

## Discussion

The wound healing process includes bleeding, inflammatory reaction, granulation tissue formation, and tissue remodeling, which is a dynamic, orderly, and interlacing process. However, this process may be destroyed under the action of various internal and external factors, resulting in the pathological inflammatory reaction, and ultimately leading to chronic refractory wounds (Takagi et al., 2020). The clinical characteristics of refractory wounds mainly include tissue defects caused by severe trauma, severe infection on the wound, impairment of microcirculation on the wound and its surroundings, and severe underlying diseases or poor nutritional status (Zhang et al., 2020). Chronic refractory wounds have slow self-repair, a long course of the disease and are easy to repeat. At the same time, chronic incurable wounds will aggravate the primary disease (Wang et al., 2019). The pathogenesis of refractory wounds is very complex, including a series of cascade reactions such as tissue ischemia and hypoxia, growth factor deficiency, apoptosis, and oxidative stress (Lu et al., 2021). In addition, the long-term unhealing of refractory wounds will lead to the aging of the granulation tissue of the wound, the formation of biofilms and fibrous plates, and the reduction of the expression of various growth factors, further delaying the wound healing process, and forming a vicious circle, which can lead to malignant transformation (Gu et al., 2021). On the other hand, long-term non-healing of refractory wounds will lead to wound granulation tissue aging, the formation of biofilm and fiberboard, the expression of various growth factors reduced, further delay the process of wound healing, and even lead to malignant transformation (Lu et al., 2021). Therefore, how to effectively treat refractory wounds has always been the focus of clinical research.

VSD is a common wound repair method currently in practice. Its principle is to use porous foam material and a biological semi-permeable membrane to turn open wounds into closed wounds, carry out continuous or intermittent attraction, and timely remove wound secretions and necrotic tissue fragments while increasing blood flow and reducing local tissue edema (Gu et al., 2021). Duan et al. (2019) reported that negative pressure wound treatment could effectively control infection, reduce the positive rate of bacteria in wound, shorten the length of hospital stay, and improve wound healing. Yang et al. (2019) suggested that VSD combined with platelet-rich plasma gel revealed a better bacteriostatic effect than negative pressure wound therapy alone in the treatment of chronic refractory wounds, which could effectively shorten the wound healing time and improve the wound healing rate. Wu et al. (2020) reported that VSD could simplify complex wounds and provide technical advantages in reducing dressing frequency, preventing wound infection, and accelerating wound healing. However, the growth and regeneration of wound granulation tissue require sufficient concentration of growth factors, control of excessive apoptosis of cells, and revascularization, the VSD technique has no advantages in these aspects (Guo et al., 2018).

Autologous blood concentrate contains a large number of platelets, growth factors, fibrin, and white blood cells, and has been widely used in the field of repair and reconstruction because of its ability to accelerate wound healing and promote regeneration of hard and soft tissues (Wang and Zhong, 2021). The i-PRF is a concentrated solution of platelet extracted from whole blood by centrifugation, including the release of a large number of growth factors and white blood cells such as lymphocytes, neutrophils, and monocytes, which can enhance The anti-infection ability of wound surface. Liu et al. (2021) pointed out that i-PRF can promote wound healing, prevent wound infection, shorten treatment time and relieve patients’ pain. Zhang et al. (2021) (Xu et al., 2020) suggested that i-PRF combined with VSD can significantly promote wound healing of refractory mastitis ulcers and shorten the wound healing time. The results of this study showed that the positive rate of bacterial culture in the study group was lower than that in the control group during follow-up (*p* = 0.011), suggesting that the combination of i-PRF and VSD has a certain degree of synergistic effect in promoting wound healing and bacterial transplantation. The reason for this result may be that i-PRF combined with VSD promotes the peripheral blood circulation and cell growth and metabolism, which is beneficial to reduce the occurrence of infection and promote the healing of chronic and refractory wounds. The process of obtaining i-PRF can be complicated and expensive and is influenced by many vendors and proprietary techniques (Gentile, 2020). In this study, the use of a simple standard low-cost method of twice-centrifugation for preparation may help standardize research protocols and permit the comparison of results from similar treatment biologics. Easy preparation may lead to more widespread use and the ability to establish clinical efficacy in various procedures.

The inflammatory response of refractory wounds is a potential therapeutic target. Previous studies have suggested that there are pathological conditions of inflammatory cell aggregation, metabolic disorder, and cell swelling in refractory wounds, which lead to tissue damage (Wang et al., 2019). The WBC, CRP, and IL-6 are common inflammatory factors Shen et al. (2020). (Aydinyurt et al., 2021) reported that the level of inflammatory factors in patients with refractory wounds was highly expressed, and effective treatment could reduce the level of inflammatory factors in patients. The results of this study suggested that the WBC, CRP, and IL-6 in the study group were lower than that in the control group on 7th day and 14th day after treatment (*p* < 0.001), suggesting that i-PRF combined with VSD could better reduce the degree of inflammation in patients. Potential mechanisms could be as follows: 1) the i-PRF contains vascular endothelial growth factor, IL-6, tumor necrosis factor –α, and other cytokines involved in inflammatory regulation, which can regulate immune response, maintain a dynamic balance between pro-inflammatory and anti-inflammatory cytokines, enhance anti-infection ability, promote angiogenesis, and facilitate wound (Jasmine et al., 2020) healing; 2) it has been reported that i-PRF is rich in platelets, antimicrobial peptides, thrombin, and other components, and interferes with the metabolic activity of bacterial cells through the action of related permeable protein, lactoferrin, and other active molecules, leading to the stage of apoptosis and necrosis, exerting strong anti-bacterial biofilm and antibacterial activity (Norman et al., 2020); 3) the VSD could timely drain wound secretions and exudate, reduce edema, establish a liquid balance microenvironment conducive to wound healing, and play a synergistic role with i-PRF to accelerate wound healing.

The scar is another therapeutic target for refractory wounds. Lin et al. (2020) reported that for patients with refractory bone exposure, the application of i-PRF combined with nano-silver antibacterial dressing could reduce the inflammatory response and restore the skin color of the wound. The results of the present study suggested that the VSS score of the study group was significantly lower than that of the control group after treatment (*p* < 0.001), suggesting that i-PRF combined with VSD could reduce the pigmentation of scars in patients with chronic refractory wound and improve the scar situation. At the same time, the present study also suggested that i-PRF combined with VSD also had a lower incidence of adverse reactions. It was considered that the injectable platelet-rich fibrin was extracted from patients’ venous blood without the risk of immune rejection and disease transmission previously faced by allogeneic growth factors, so the incidence of adverse reactions could be reduced.

This study also had the following limitations: 1) Since the study duration was relatively short, the follow-up duration should be extended in the later stage study; 2) At present, there is no unified standard for the preparation of i-PRF, and more studies are needed to compare i-PRF prepared by different separation methods; 3) It would be better to single out groups as venous ulcers, arterial ulcers, and diabetic ulcers.

## Conclusion

This study suggested that i-PRF combined with VSD could accelerate the healing of chronic refractory wounds and improve the wound healing rate. Compared with VSD alone, combining i-PRF and VSD revealed more advantages in reducing serum inflammation levels and positive rate of bacterial culture, as well as improving scar healing.

## Data Availability

The original contributions presented in the study are included in the article/supplementary material, further inquiries can be directed to the corresponding author.
